# Photosensitizer-singlet oxygen sensor conjugated silica nanoparticles for photodynamic therapy and bioimaging†

**DOI:** 10.1039/d3sc03877g

**Published:** 2023-12-14

**Authors:** Jeladhara Sobhanan, Kenji Ono, Takuya Okamoto, Makoto Sawada, Paul S. Weiss, Vasudevanpillai Biju

**Affiliations:** a Graduate School of Environmental Science, Hokkaido University Sapporo Hokkaido 060-0810 Japan biju@es.hokudai.ac.jp; b Department of Chemistry, Rice University Houston Texas 77005 USA; c Research Institute of Environmental Medicine, Nagoya University Nagoya 464-8601 Japan; d Research Institute for Electronic Science, Hokkaido University Sapporo Hokkaido 001-0020 Japan; e California NanoSystems Institute and the Departments of Chemistry and Biochemistry, Bioengineering, and Materials Science and Engineering, University of California Los Angeles CA 90095-1487 USA

## Abstract

Intracellular singlet oxygen (^1^O_2_) generation and detection help optimize the outcome of photodynamic therapy (PDT). Theranostics programmed for on-demand phototriggered ^1^O_2_ release and bioimaging have great potential to transform PDT. We demonstrate an ultrasensitive fluorescence turn-on sensor-sensitizer-RGD peptide-silica nanoarchitecture and its ^1^O_2_ generation–releasing–storing–sensing properties at the single-particle level or in living cells. The sensor and sensitizer in the nanoarchitecture are an aminomethyl anthracene (AMA)-coumarin dyad and a porphyrin or CdSe/ZnS quantum dots (QDs), respectively. The AMA in the dyad quantitatively quenches the fluorescence of coumarin by intramolecular electron transfer, the porphyrin or QD moiety generates ^1^O_2,_ and the RGD peptide facilitates intracellular delivery. The small size, below 200 nm, as verified by scanning electron microscopy and differential light scattering measurements, of the architecture within the ^1^O_2_ diffusion length enables fast and efficient intracellular fluorescence switching by the tandem ultraviolet (UV)-visible or visible-near-infrared (NIR) photo-triggering. While the red emission and ^1^O_2_ generation by the porphyrin are continually turned on, the blue emission of coumarin is uncaged into 230-fold intensity enhancement by on-demand photo-triggering. The ^1^O_2_ production and release by the nanoarchitecture enable spectro-temporally controlled cell imaging and apoptotic cell death; the latter is verified from cytotoxic data under dark and phototriggering conditions. Furthermore, the bioimaging potential of the TCPP-based nanoarchitecture is examined *in vivo* in B6 mice.

## Abstract 2

Intracellular singlet oxygen (^1^O_2_) generation and detection help optimize the outcome of photodynamic therapy (PDT).

## Graphical abstract

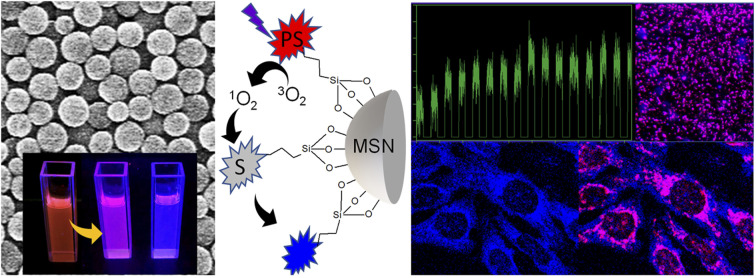

## Introduction

Reactive oxygen species (ROS)-mediated photodynamic therapy (PDT) is a promising tool in cancer management. The unique oxidizing power of metastable singlet oxygen (^1^O_2_) received considerable attention in synthetic chemistry, environmental remediation, and therapeutics.^1–3^ Notably, ^1^O_2_ released by photosensitizer (PS) drugs triggers the disruption of the tumor microvasculature, oxidative stress-induced cellular damage, and impaired membrane transport functions.^4–6^ The cytotoxic ^1^O_2_ produced by PS drugs precisely delivered in a tumor milieu avoids or minimizes undesired toxicity to normal cells and tissues due to systemic overdose or nonspecific drug localization. Furthermore, the nonspecific toxicity of PS drugs is minimized by local phototriggering. Therefore, the detection and quantification of ^1^O_2_ produced by a PS drug at the desired location are of utmost significance in PDT. An array of fluorescence (FL) probes built on polycyclic and heterocyclic aromatics has been developed to detect or sense ^1^O_2_.^7–12^ However, the performances of such probes are limited due to inadequate FL turn-on features, low sensitivity, and complex syntheses. Nevertheless, anthracene derivatives dominate as safe ^1^O_2_ sensors and carriers, and substituents at the 9,10-positions of anthracene modulate the ^1^O_2_ sensitivity.^13–17^ In addition, the self-limiting factor of ^1^O_2_ invokes the hypoxic microenvironment in tumors and thus proliferates the cancer metastasis or builds up a resistance towards PDT.^18–22^ Therefore, localized generation, controlled supply, and accurate detection of ^1^O_2_ using probes with high spectroscopic/microscopic sensitivity are critical for advancing PDT. The co-existence of ^1^O_2_ sensing and controlled releasing using a sensor-sensitizer system would help overcome the challenges associated with photooxygenation techniques.^23–26^

Nanomaterials offer myriad advantages over traditional drug delivery systems (DDS). Various photoresponsive, biocompatible nanomaterials have been recently developed for this purpose.^27–33^ Mesoporous silica nanoparticles (MSN) are among the most promising theranostic platforms for cancer imaging and therapy.^34–44^ Caged drugs/contrast agents in MSN by chemical functionalization protect their intrinsic properties, promote aqueous solubility, and increase tumoral accumulation.^34–43^ Also, the mesoporous structure and high surface-to-volume ratios of MSNs increase the drug/contrast agent loading and delivery efficiencies.^42,43^ Recently, Meng and Nel developed lipid bilayer-coated MSN (silicasomes) for intratumoral delivery of chemotherapeutics such as oxaliplatin, gemcitabine, or paclitaxel to the Kras-derived pancreatic cancer in mice.^38,39^ Similarly, they studied the antitumor effects of irinotecan-loaded silicasomes in pancreatic and colorectal cancers, utilizing the ability of irinotecan to neutralize the lysosomal acidity.^40,41^ Likewise, MSN-coupled ^1^O_2_ FL probes are widely applied in PDT. For example, Jiao *et al.* prepared a silica nanocarrier (FSNC) functionalized with the PS protoporphyrin IX, a 2-pyridone derivative, as the ^1^O_2_ storage/release unit, and a cyanine derivative as the ^1^O_2_ self-monitoring unit for fractional PDT.^45^ Under light-induced ^1^O_2_ generation, the FSNC forms endoperoxide and releases the stored ^1^O_2_ under the dark by a cycloreversion mechanism, monitored continuously by the FL bleaching of the cyanine dye. Also, they demonstrated the biocompatibility and bioimaging potentials of NIR-dyes covalently incorporated in silica nanoparticles.^46^ In another report, they constructed hydrophobic domains in nanosilica using a modified silane coupling agent and preserved the FL of NIR dyes by preventing aggregation.^47^ A polymeric nanocarrier for enhanced phototherapy is another example.^48^ Here, dual NIR laser-regulated ^1^O_2_ trapping by an anthracene-BODIPY conjugate enabled the sustained ^1^O_2_ release. Also, Stang and coworkers developed an organoplatinum(ii) supramolecular metallacycle by coordination-driven self-assembly of dipyridyl anthracene donor – Pt(ii) acceptor for reversible ^1^O_2_ capture and release, which showed high photooxygenation and thermolysis rates.^49^ Nonetheless, programmed nanomaterials integrating multiple fluorescent PS drugs and fluorogenic sensors for ^1^O_2_ storing, sensing, and controlled releasing from a single framework are highly sought after for next-generation PDT.

Here, we describe and demonstrate a sensor-sensitizer-based multifunctional mesoporous silica nanoarchitecture and its on-demand ability to produce, to store, to release, and to sense ^1^O_2_ continuously at the single-particle level and in living cells. The nanoscale (200 nm diameter) silica and multiple sensor–sensitizer conjugates within the diffusion length of ^1^O_2_ in cells enable efficient ^1^O_2_ storing–sensing–releasing even at the single-cell and single-particle levels. Here, a porphyrin [tetrakis(4-carboxyphenyl)porphyrin (TCPP)] and an uncaged coumarin provide the spectrally and visibly distinct bimodal FL to the nanoarchitecture. Indeed, the blue FL is enormously increased (230-fold) with time under photosensitization of the PS. The FL of the coumarin in the nanoarchitecture is quantitatively quenched by lock-in photoinduced intramolecular electron transfer (PIET), which is nearly 100% efficient from an aminomethyl anthracene (AMA). This efficient PIET enables the highest ^1^O_2_ sensitivity to the sensor molecule or the nanoarchitecture. The FL intensity enhancement by the one- (UV-vis) or two- (NIR) photon-triggered uncaging (releasing coumarin FL by oxidation of the AMA part) represents ^1^O_2_ sensing. Solution-based, single-particle and single-cell experiments demonstrate outstanding ^1^O_2_-induced FL turn-on efficiency at a controlled rate for sensor 4 compared to previously reported sensors such as Si-DMA (*ca.* 10-fold),^50^ or SOSG (*ca.* 50-fold).^51^ Furthermore, an arginine-rich peptide conjugate facilitates cellular uptake of the nanoarchitecture. Along with the intracellular production and release of ^1^O_2_ by the nanoarchitecture, the colocalized red FL of the PS and the intense blue FL of the uncaged sensor offer spectrally-resolved imaging and PDT modalities. The cytotoxicity of the nanoarchitecture is evaluated by MTT [3-(4,5-dimethylthiazol-2-yl)-2,5-diphenyltetrazolium bromide] assay in MCF7 cells under dark and photo-triggering conditions, which showed significant phototoxicity. We selected TCPP considering its carboxylic groups for functionalization and its photophysical properties, such as broad absorption in the UV-vis region, deep red FL (>650 nm), and ^1^O_2_ production (10% in water and 4.3% in DMF).^52^ We prepared a nanoarchitecture analogous to the TCPP-based one using near-infrared (NIR) emitting CdSe/ZnS quantum dots (QD) instead of TCPP, considering the wealth of information about QDs for bioimaging.^53–58^ Also, we considered the ability of the QDs to generate ^1^O_2_.^56–58^ Further, we examined the *in vivo* FL imaging potential of the SS-MSNP-RGD nanoarchitecture by applying it to B6 mice intravenously or subcutaneously.

## Experimental section

### Materials and methods

All chemicals used in this research were analytical grade and used as received unless otherwise stated. Silica nanoparticles and (+) biotin-*N*-hydroxysuccinimide ester (biotin-NHS) were obtained from Sigma Aldrich. Potassium carbonate (K_2_CO_3_) and 4-(4,6-dimethoxy-1,3,5-triazin-2-yl)-4-methylmorpholinium chloride (DMT-MM) were obtained from FUJIFILM Wako Pure Chemical Corporation, Japan. 7-Amino-4-methyl coumarin, *N*,*N*-dioctylamine, 9,10-bis(chloromethyl)anthracene, and TCPP were obtained from Tokyo Chemical Industry (TCI), Japan. Qdot™ 655 (QD655)-streptavidin conjugate, fetal bovine serum (FBS), penicillin/streptomycin (P/S), and trypsin were obtained from ThermoFisher Scientific, USA. RGD-SH (CDCRGDCFC) was purchased from GenScript, Japan. The MTT assay kit was obtained from Rosche (Sigma-Aldrich) and used as described by the manufacturer. Optical densities of MTT samples were measured using a microplate reader (Multiskan Sky TCD, ThermoFisher, USA). All solvents were reagent grade from FUJIFILM Wako Pure Chemical Corporation, Japan.

**Fig. 1:**
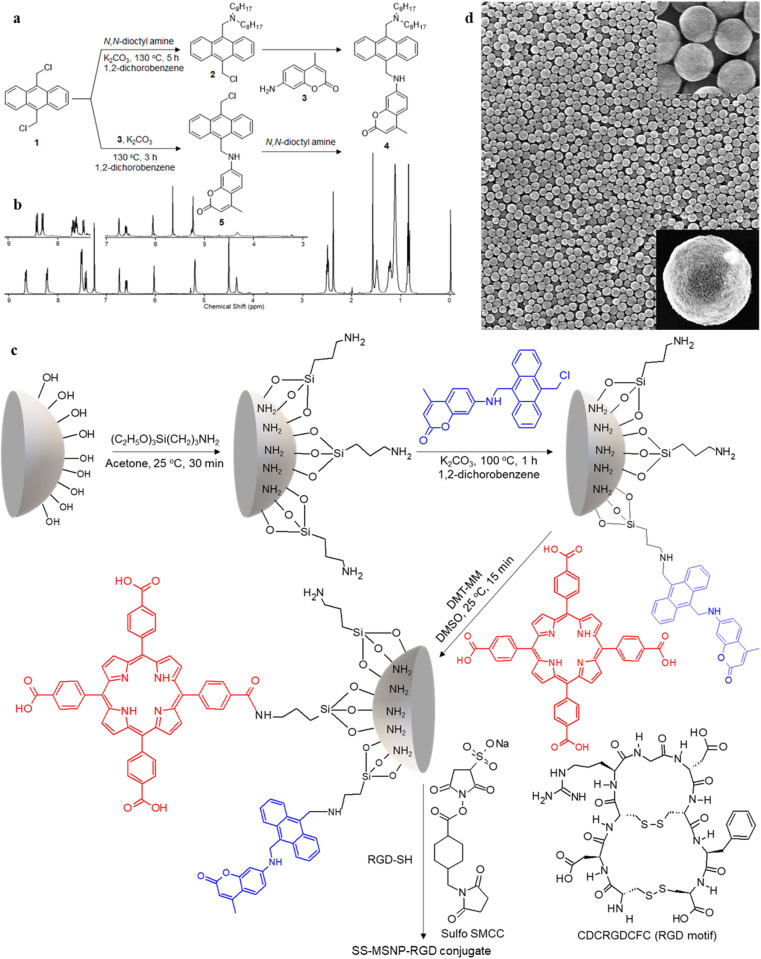
Synthesis of the sensor and sensor-sensitizer-silica nanoarchitectures (SS-MSNPs). A scheme showing the synthesis of (a) sensor 4 and the intermediate 5, and (c) S-MSNP and SS-MSNP conjugates. The structures of the amine-to-thiol cross-linker (Sulfo SMCC), and RGD-SH and its sequence are also shown in ‘c’. (b) ^1^H NMR spectra of sensor 4, and the intermediate 5 (inset), and (d) scanning electron microscopy images of MSNPs before (low magnification) and after surface modification (inset).

Absorption spectra were recorded using a UV-visible spectrophotometer (Evolution 220, ThermoFisher Scientific), and FL spectra were recorded using a Hitachi F4500 Spectrofluorometer. Nuclear magnetic resonance (NMR) measurements were performed in a JEOL 400 MHz NMR spectrometer. A diode-pumped solid-state (DPSS) 532 nm green laser (Spectraphysics or Shanghai Dream Laser Technology) or a mercury vapor lamp (365 nm) was used for the photoirradiation experiment at the single-particle or ensemble levels. The scanning electron microscope (SEM) images (Fig. 1d) were recorded in a Hitachi HD-2000 microscope operated at 200 kV. Microtoming of cell samples was carried out using an ultramicrotome EM UC7i (Leica-microsystems), and TEM images of the microtomed samples were recorded at 25 kV on a Hitachi field emission (FE) SEM (SU8230) equipped with a STEM detecter. Zeta potentials of the samples were measured in an ELS-Z 2 system (Otsuka Electronics Co., Ltd.). Dynamic light scattering (DLS) experiments were conducted on an FDLS-3000 system (Otsuka Electronics Co., Ltd.) FL images of the B6 mice were obtained using a Maestro small animal imaging system (PerkinElmer).

### Synthesis and characterization

#### Synthesis of sensor 4

9,10-Bis(chloromethyl)anthracene 1 (1.38 g, 5.01 mmol) was dissolved in 1,2-dichlorobenzene (20 mL) at 130 °C followed by the addition of *N*,*N*-dioctylamine (0.96 g, 3.97 mmol). Then, K_2_CO_3_ (1.36 g, 9.84 mmol) was added to the solution, and the mixture was stirred at 130 °C for five hours under the argon atmosphere, during which the intermediate (2) was formed. Then, 7-amino-4-methylcoumarin 3 (680 mg, 3.88 mmol) was added to the solution, and the stirring was continued for 12 h. Afterward, the reaction mixture was cooled to room temperature, and the residue formed was removed by filtration. The filtrate was collected, and the solvent was removed by vacuum distillation. The product was purified by silica-gel column chromatography using 10% ethyl acetate and hexane mixture, giving a pale-yellow solid powder (50 mg, 35%). The steps involved in the synthesis are shown in Fig. 1a. We optimized this yield by repeating the synthesis five times.

^1^H NMR (400 MHz, CDCl_3_, Fig. 1b): *δ* = 8.64–8.67 (d, 2H, Ar–H), 8.21–8.23 (d, 2H, Ar–H), 7.51–7.53 (m, 4H, Ar–H), 7.42–7.45 (d, 1H, Ar–H), 6.75 (s, 1H, Ar–H), 6.60–6.62 (d, 1H, Ar–H), 6.04 (s, 1H, allylic), 5.21 (d, 2H, CH_2_–NH), 4.52 (s, 2H, Ar–CH_2_–N), 4.36 (t, 1H, NH), 2.51–2.53 (t, 4H, N–CH_2_), 2.39 (s, 3H, CH_3_), 1.4–1.55 (q, 4H, N–CH_2_–C), 1–1.35 (m, 2H, C–CH_2_–C), 0.5–0.8 (t, 6H, C–CH_3_). ^13^C NMR (100 MHz, THF-D_8_) 14.63, 18.53, 23.73, 28.09, 28.61, 30.49, 33.00, 41.37, 52.39, 54.49, 98.30, 109.99, 110.78, 125.76, 125.94, 127.72, 127.04, 130.38, 131.51, 132.47, 133.36, 153.14, 153.55, 157.70, 161.05. HR-ESI-MS *m*/*z* for C_42_H_54_O_2_N_2_ – [M^+^] – calcd: 618.8971 found: 619.4229.

#### Synthesis of 5

9,10-Bis(chloromethyl)anthracene 1 (2 g, 7.26 mmol) was dissolved in 1,2-dichlorobenzene (30 mL) at 130 °C followed by the addition of 7-amino-4-methylcoumarin 3 (0.87 g, 4.99 mmol). Then, K_2_CO_3_ (1.36 g, 9.84 mmol) was added to the solution, and the mixture was stirred at 130 °C for three hours under the argon atmosphere. Afterward, an excess of hexane was added to precipitate the product, which was collected by filtration. The purification of the crude product is carried out by repeated precipitation with toluene and acetonitrile, which provided (0.1 g, 45%) 5. We optimized this yield by repeating the synthesis five times. Similar to the above synthesis, 4 was obtained by the subsequent reaction of 5 with *N*,*N*-dioctylamine. The steps involved in the synthesis are shown in Fig. 1a.

^1^H NMR (400 MHz, CDCl_3_, Fig. 1b): *δ* = 8.41–8.43 (d, 2H, Ar–H), 8.29–8.43 (d, 2H, Ar–H), 7.62–7.67 (m, 4H, Ar–H), 7.44–7.46 (d, 1H, Ar–H), 6.76 (s, 1H, Ar–H), 6.61–6.63 (d, 1H, Ar–H), 6.06 (s, 1H, allylic), 5.66 (s, 2H, CH_2_–Cl), 5.24 (s, 2H, CH_2_–NH), 4.33 (s, 1H, NH). ^13^C NMR (400 MHz, CDCl_3_): *δ* = 18.72, 31.03, 38.97, 40.72, 98.18, 110.02, 110.46, 111.18, 124.42, 124.76, 125.80, 126.79, 126.89, 128.32, 129.13, 129.67, 129.84, 130.38, 130.48, 151.21, 153.01, 156.20, 161.97. ^13^C NMR NMR (100 MHz, DMSO-D_6_) 17.89, 96.41, 107.46, 108.88, 110.24, 124.34, 125.04, 125.90, 126.13, 126.42, 129.08, 129.78, 131.32, 133.60, 152.18, 153.58, 155.53, 160.60.

#### Preparation of S-MSNP and SS-MSNP

Mesoporous silica nanoparticles (Si NPs, 0.5 g, 200 nm size, Fig. 1d) were silanized by adding 25 μL APTES solution (1 wt% APTES, 80 wt% acetone, 19 wt% water). The solution was stirred for 30 min at 25 °C. The settled particles were thoroughly rinsed with water and acetone and dried. Then, a solution of 5 (5 mM) in 1,2-dichlorobenzene was added to the amino-functionalized MSNP and stirred for one hour at 100 °C in the presence of K_2_CO_3_ (0.5 mg, 3.61 μmol). After one hour, the supernatant was collected by centrifugation, and the number of 5 reacted per MSNP was determined from the number of silica particles (based on the weight, specific gravity, and average diameter) and the difference in the absorbencies of the solutions of 5 before and after the reaction. The particles were thoroughly washed with acetone and water and dried to obtain the silica–sensor conjugate S-MSNP (Fig. 1c). To prepare the silica-sensor-TCPP conjugate SS-MSNP, a TCPP solution (500 μM) in dimethylsulfoxide (DMSO) was added to the S-MSNP and stirred for 15 min in the presence of DMT-MM (0.13 mg, 0.46 μmol) as the coupling agent at 25 °C. The resultant sample was thoroughly washed with methanol and DMSO and dried. We estimated the number of TCPP molecules per SS-MSNP from the difference absorption spectrum by following the method for S-MSNP. For the conjugation of a cell-penetrating ligand, the free primary amino groups in the SS-MSNP (5 mg) were reacted with the –NHS ester part of the heterobifunctional cross-linker sulfo-SMCC (Fig. 1c, 2.3 mM) in phosphate-buffered saline (PBS) for 30 min at 25 °C. The particles were thoroughly washed to remove the free sulfo-SMCC and were collected by centrifugation. An RGD-SH (CDCRGDCFC) peptide solution (1.33 mM) in PBS was added to the SS-MSNP particles, and the maleimide reactive group of the cross-linker on the SS-MSNP particles was allowed to react with the peptide for one hour. Finally, the particles were repeatedly washed with PBS and resuspended in PBS. Although the S-MSNP and SS-MSNP particles showed decreased suspension in water, the amino- or RGD-functionalized particles showed excellent aqueous phase suspension than the original MSNPs, which is attributed to the hydrophilic and proton buffering properties of the primary amino group and the peptide.

#### Preparation of the sensor-QD-MSNP (S-QD-MSNP) nanoarchitecture

To the S-MSNP conjugate prepared previously, a biotin-NHS ester solution (1 mM) in DMSO was added and stirred for four hours at 25 °C to introduce biotin units. The particles were thoroughly washed with water and acetone. In the next step, a QD655-streptavidin solution (10 nM) in PBS was added, reacted for 30 min, centrifuged, washed with PBS, and resuspended in water. These NPs were also labeled with the RGD peptide, as stated above.

#### Steady-state absorption and FL spectroscopic measurements

A sample solution of a sensor 4 (10 μM) and TCPP (5 μM) in CH_3_CN was photosensitized using a 532 nm laser (50 mW cm^−2^) for 60 min followed by UV lamp illumination (365 nm, 1 mW cm^−2^) for 120 min. The corresponding absorption and FL spectra (*λ*_ex_ = 320 nm) were recorded before and after the photoactivation. Similarly, 532 nm photoactivation of the S-MSNP (15 mg) in a 5 μM TCPP solution in DMF, SS-MSNP (15 mg) in DMF, and S-QD-MSNP (15 mg) in PBS was carried out for 60 min. The corresponding absorption and FL spectra (*λ*_ex_ = 320 nm) were also recorded.

#### Single-particle FL measurements

Single-particle samples were prepared by placing the sample suspensions (1 mL) on glass coverslips (25 × 50 mm^2^). The FL intensity trajectories of the S-MSNP attached to the coverslip and immersed in a TCPP solution or the SS-MSNP conjugate attached to the coverslip were recorded on a microscope (IX70, Olympus) equipped with an EMCCD (iXon, Andor Technology) camera, a 40× Olympus objective lens (NA = 0.60), a 420–480 nm band-pass (BP) filter (for coumarin) and a 600 nm long-pass (LP) filter (for TCPP). The sensitizer in the samples was photoactivated with a 532 nm laser (Millenia IIs, Spectra-Physics, 5 W cm^−2^) at 30 s intervals followed by UV illumination (320 to 390 nm) for 2 min, during which the FL images, videos, and intensity trajectories were recorded through the BP filter for coumarin. Similarly, the FL images, videos, and intensity trajectories were recorded for S-QD-MSNP samples with 1 min photoactivation (532 nm) and 2 min UV illumination (320 to 390 nm). The FL intensity trajectories at different power densities were collected and analyzed. Single particle experiments were repeated for three samples in each case, each >100 particles.

#### Confocal laser scanning microscopy (CLSM)

Human MCF7 adenocarcinoma breast cells (∼60% confluency) were cultured in DMEM supplemented with 10% heat-inactivated FBS and 1% P/S under a humidified atmosphere with 5% CO_2_ and at 37 °C. MCF7 cells (TKG0479, Cell Resource Center for Biomedical Research, Tohoku University) were obtained from the Riken Cell Bank (RCB1904). For cell imaging, the cells were incubated with SYTO-16 nucleus staining dye (5 μM) for 15 min and washed with PBS. Next, the cells were incubated with different silica samples (50 μg mL^−1^) in a serum-free medium for 30 min, followed by PBS washing. The FL images of the labeled cells were collected using a CLSM system (Nikon Ti2, Nikon Corporation, Japan) equipped with an oil-immersion objective lens (PlanApo VC 60×/NA. 1.40). The cell samples were excited with 402 nm, 488 nm, 561 nm laser with the FL detection channels at 420–490 nm (sensor), 520–560 nm (SYTO-16), 610–730 nm (TCPP), and 630–690 nm (QD655), respectively. Cell labeling and imaging were repeated six times at 60% cell confluency.

#### Cytotoxicity assays

We examined the dark cytotoxicity and photocytotoxicity of the nanoarchitectures to MCF7 cells. The cells cultured in 96-well microplates for two days in DMEM medium were washed with PBS and treated with the nanoarchitecture samples dispersed in PBS for 30 min at 36 °C. Unlabeled NPs were removed by washing using PBS. Next, serum-containing DMEM medium was added to the cells, and the cells were maintained in the dark for 30 min and were treated with MTT by following the MTT assay protocol by the manufacturer. The MTT-treated cells were incubated at 36 °C overnight, washed with PBS, and treated with the cell lysis medium for 6 h. After dissolving the formazan formed inside the cells, the optical densities of the samples were measured, and the cell viability values were calculated for three sets of each sample using a microplate reader.

#### *In vivo* experiments

*In vivo* imaging experiments were carried out using B6 mice. Six male mice were used in this study. Each mouse was anesthetized by inhalation of 1% isoflurane. Hair removal on all anesthetized mice was performed from the neck to the lower legs with a depilatory cream. Three mice were intravenously or subcutaneously injected with different samples (S-MSNP, Silica-porphyrin, or SS-MSNP-RGD). FL images were taken at 1 min, 1 h, and 24 h post-injection using a Maestro imaging system equipped with a blue filter set (*λ*_ex_ = 445–490 nm and *λ*_em_ > 515 nm). FL signals were extracted from the images using suitable filters and analyzed using the Maestro software. These experiments were conducted after approval (approval number: R220005) from the Nagoya University Care and Use of Animals Committee (constituted on 24 March 2022), which are strictly under the animal experiment guidelines of Nagoya University.

## Results and discussion

### ^1^O_2_ generation and sensing in solutions

The ^1^O_2_ generation, storage, sensing, and delivery efficiencies of the nanoarchitecture samples are based on highly efficient PIET from the bis-AMA to coumarin in sensor 4 (Fig. 1a). Sensor 4 was synthesized in one pot by a sequential base-catalyzed nucleophilic substitution reaction between 9,10-bis(chloromethyl)anthracene (1), 7-amino-4-methylcoumarin (3) and *N*,*N*-dioctylamine. The bis AMA group is critical in the quantitative electron transfer-induced FL quenching and 230-fold FL intensity enhancement during the sensing. Conversely, the initial coumarin FL was not efficiently quenched in the corresponding intermediate (5). Therefore, it does not show the ^1^O_2_ sensitivity like 4 because of poor PIET from the chloromethyl anthracene to coumarine parts. The nanoarchitecture integrated with a ^1^O_2_ sensor and a suitable PS, such as TCPP or QDs, overcomes various limitations in conventional PDT.^34–37^ The red FL from TCPP or QD and the enhanced blue emission from the sensor offer ^1^O_2_ sensing and two-color imaging modalities during PDT. To develop SS-MSNP, the intermediate 5 and TCPP or 5 and QD were attached covalently to the amino-functionalized MSNPs (Fig. 1a and c). The covalent attachment prevents the undesired sensor/sensitizer release or leaching, thereby maintaining photoactivity.^59–65^ We selected MSNPs considering the advantages of mesopores for efficiently loading drugs and contrast agents. Nevertheless, directly incorporating sensors or sensitizers in nonporous silica during NP synthesis^45–47^ does not limit the current work. Fig. 1d shows the SEM images of the functionalized MSNPs. The particles showed a uniformly distributed spherical morphology with a 200 nm average diameter. The S-MSNP, SS-MSNP, and SS-MSNP-RGD samples were further characterized by zeta potentials (+16.91 mV for S-MNSP, +15.52 mV for SS-MSNP, and −15.02 for SS-MSNP-RGD), and DLS measurements (the size distributions are shown in Fig. S1†).

**Fig. 2:**
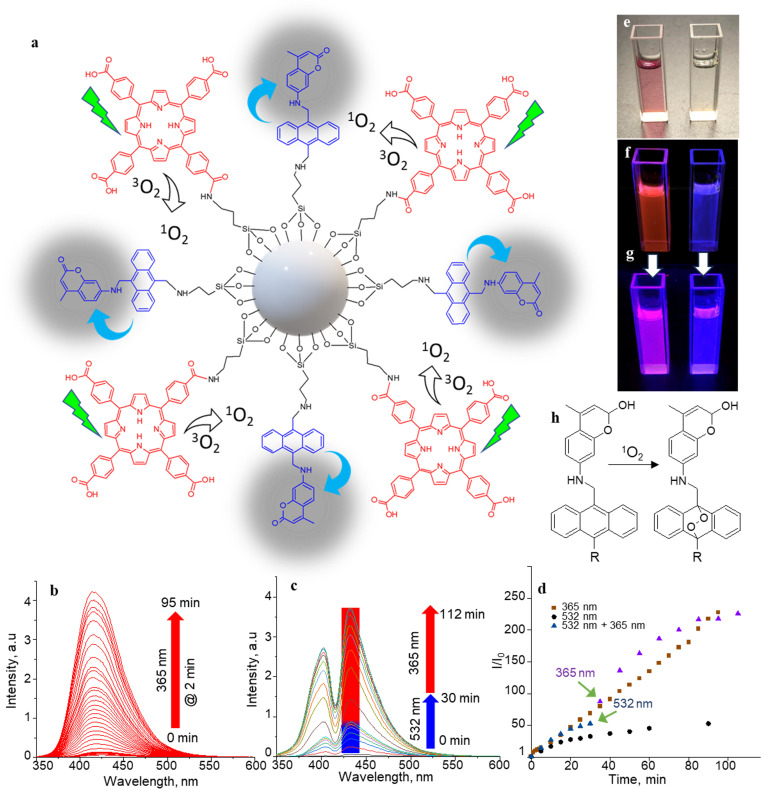
^1^O_2_ generation, sensing, and releasing. (a) The SS-MSNP structure and its ^1^O_2_ generation and sensing processes. (b and c) FL spectra (*λ*_ex_ = 320 nm) of (b) 4 (10 μM in CH_3_CN, without TCPP) before and after irradiation at 365 nm for 95 min at 2 min intervals, (c) 4 (10 μM in CH_3_CN) in a TCPP solution (5 μM) before and after photoactivation at 532 nm (50 mW cm^−2^) for 30 min (blue bar) followed by UV illumination (365 nm, 5 mW cm^−2^) for 82 min (red bar) showing the sensitization-sensing process followed by UV-induced ^1^O_2_ releasing/AMA oxidation. (d) Time-trace of the peak FL intensities at different photoirradiation conditions in (b) and (c). (e–g) Photographs of (left) a mixture of 4 and TCPP and (right) 4 (e) under visible light before photoactivation, (f) <1 min under UV light after 30 min 532 nm laser irradiation (50 mW cm^−2^) for 30 min, and (g) *ca.* 5 min under UV light after 30 min 532 nm laser irradiation (50 mW cm^−2^). (h) A scheme of ^1^O_2_ reaction on the sensor.

The pivotal role of 4 in S-MSNP and SS-MSNP as an excellent ^1^O_2_ sensor and donor was investigated by measuring its photophysical properties at the ensemble and single-particle levels. Fig. 2a shows the photosensitization and ^1^O_2_ sensing processes on an SS-MSNP similar photochemical processes take place in a solution of 4 and TCPP. First, we examined the FL spectra of 4 under UV light irradiation without and with ^1^O_2_ scavengers (NaN_3_ or CuCl_2_) to distinguish between the roles of UV-induced anthracene dimerization^66^ and ^1^O_2_-mediated anthracene oxidation in blocking PIET from AMA to coumarin. The UV-induced FL intensity enhancement of 4, without any ^1^O_2_ scavenger, exceeded 230-fold (Fig. 2b and d). Conversely, the FL intensity enhancement was <60-fold under UV irradiation (Fig. S2†) in the presence of NaN_3_ or CuCl_2_. Therefore, we assume 4 generates ^1^O_2_ under UV irradiation, and AMA oxidation by ^1^O_2_ blocks PIET and enhances the FL intensity by >170-fold. The >170-fold enhancement factor includes the FL of the ^1^O_2_-4 intermediate and coumarin released after UV-triggered oxidation of AMA. Nevertheless, unlike visible light absorbing ^1^O_2_ sensors such as Si-DMA^50^ or SOSG,^51^4 is highly stable under room light.

The selectivity of 4 to ^1^O_2_ was determined by irradiating a solution of 4 (10 μM) and TCPP (5 μM) in acetonitrile with 532 nm laser (50 mW cm^−2^). Here, TCPP generates ^1^O_2,_ and the AMA moiety in the sensor scavenges the ^1^O_2_, forming a mixture of highly fluorescent 9,10-endoperoxide^67,68^ and a less-fluorescent intermediate that further transforms into the endoperoxide on-demand by UV or NIR triggering. In this two-step process, the initial FL intensity enhancement (under 532 nm photosensitization of TCPP) was determined at 50-fold (Fig. 2c, d and S3†), with an overall enhancement of 230-fold under UV illumination, showing a 78 : 22 ratio between the intermediate and the endoperoxide. We estimated this ratio from the maximum intensity enhancement factor (∼230-fold), the FL quantum yield of the parent coumarin (0.63), and the inflation of the FL intensity by changing the photoirradiation from 532 to 365 nm. The endoperoxide formation (Fig. 2h) follows the thermal cleavage of the O–O and C–C bonds common to alkyl-substituted anthracenes.^69^ The decay of the anthracene vibronic bands at *ca.* 356, 376, and 396 nm and the corresponding increase in the intensity at *ca.* 325 nm in the absorption spectra (Fig. S4†) suggest ^1^O_2_-mediated photo-oxidation of anthracene and the formation of anthraquinone. The FL turn-on ability (230-fold) of the sensor is the highest compared to ^1^O_2_ probes reported to date, such as SiDMA^50^ and SOSG,^51^ which is due to the efficient PIET and FL quenching in 4 favored by the bis aminomethyl moiety introduced in anthracene.

Two-step vis-UV-induced FL intensity enhancement is further confirmed from the UV-triggered, time-dependent FL images of a solution containing 4 with or without TCPP. A solution of 4 with TCPP produced ^1^O_2_, whereas 4 without TCPP was the negative control (without ^1^O_2_ generation). These samples help validate the efficiency of 4 in trapping the ^1^O_2_ produced, releasing it into a solution or undergoing oxidation. The ^1^O_2_ generated by TCPP is captured and released by the sensor as indicated by the brilliant magenta emission (^1^O_2_-positive) – a combination of the TCPP red emission and the uncaged sensor's brilliant blue emission. Conversely, no color change was observed for the negative control sample under 532 nm photoirradiation. However, intense blue FL was observed from the negative control sample under prolonged UV-only irradiation (Fig. 2e–g). Like 4, the S-MSNP nanoarchitecture showed excellent ^1^O_2_ caging and releasing- or oxidation-induced FL intensity enhancement in the heterogeneous solution phase with free TCPP in the solution. Similarly, enormous FL intensity enhancement was detected for the SS-MSNP with the sensor and sensitizer covalently on the particles (Fig. S5†). The time-traced FL intensity values reveal that the intraparticle ^1^O_2_ photogeneration-capturing-releasing by SS-MSNP is more efficient than S-MSNP dispersed in a TCPP solution.

### ^1^O_2_ generation and sensing at the single particle level

**Fig. 3:**
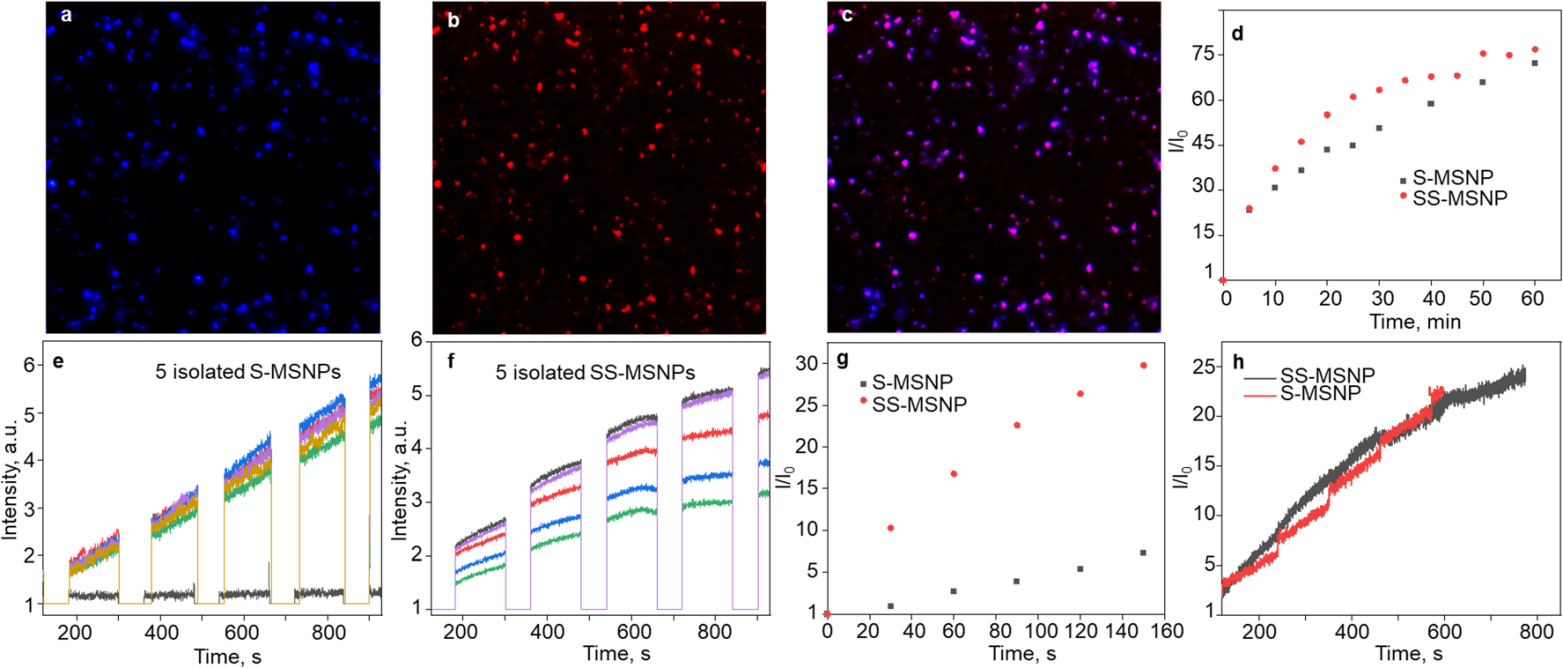
Single-particle ^1^O_2_ generation-storing-sensing-releasing properties of S-MSNPs and SS-MSNPs. (a and b) Single particle FL images (100 μm × 100 μm) of SS-MSNPs collected using (a) 420–480 nm band-pass filter, (b) 580 nm LP filter, and (c) the overlay of (a) and (b). Excitation wavelengths: (a) 365 nm (5 mW cm^−2^), (b) 532 nm (30 mW cm^−2^). (d) Time-trace of the peak (440 nm) FL intensities of the S-MSNPs in a TCPP solution (5 μM) and SS-MSNPs in water before and after photoactivation at 532 nm (5 W cm^−2^) for 60 min. (e and f) Single-particle FL intensity trajectories showing ^1^O_2_ production detected for (e) S-MSNPs in a TCPP solution and (f) SS-MSNPs in water under photoactivation of TCPP with 532 nm laser irradiation for 30 s (photosensitization) followed by UV illumination (0.5 W cm^−2^; FL releasing and detection for 120 s intervals). (g and h) Difference FL intensity profiles of S-MSNP single particles in a TCPP solution and SS-MSNP single particles in water separated into the FL intensity traces during (g) photosensitization-induced ^1^O_2_ generation and detection and (h) UV-induced ^1^O_2_ releasing and detection. A 60 s shutter temporally separated the sensitization and ^1^O_2_ release.

Single-particle FL studies help understand the photostability of S-MSNP and SS-MSNP and the microscopic-level kinetics of ^1^O_2_ generation and sensing for cell biological applications. Therefore, we examined the ^1^O_2_ generation, storing, sensing, and releasing efficiencies of the S-MSNP and SS-MSNP by recording single-particle FL images and intensity trajectories. FL images of SS-MSNP particles dispersed on a cover glass and resolved into the sensor's and sensitizer's FL are shown in Fig. 3a–c. The as-prepared SS-MSNPs exhibited intense red emission of TCPP, detected through a 620 nm LP filter by exciting at 532 nm (Fig. 3b). Conversely, the initial blue FL at *ca.* 440 nm from SS-MSNP or S-MSNP, corresponding to the coumarin moiety, was below the detection limit without the electron multiplication mode of an EMCCD camera. This is due to the FL quenching by PIET in 4. However, the FL intensity of S-MSNP and SS-MSNP was constantly increased with continuous 365 nm excitation, consistent with the FL uncaging of the intermediate.

The ^1^O_2_ capturing-releasing efficiency of the sensors in S-MSNP dispersed in a TCPP solution (Fig. 3d and e) or SS-MSNP (Fig. 3d and f) in water was examined by FL enhancement kinetic measurements of single particles. Single particle photosensitization (^1^O_2_ generation and storing) and UV-induced ^1^O_2_ release were carried out in 30–120 s sequences, as shown in Fig. 3e and f. Following the photosensitization of TCPP in an S-MSNP-TCPP solution or on the SS-MSNP surface by 532 nm laser excitation, the UV-light triggered time-dependent ^1^O_2_ releasing was detected by exciting the samples with 365 nm light (Fig. 3d). The excitation powers for photosensitization (5 W cm^−2^) and UV-induced FL uncaging (0.5 W cm^−2^) were set at higher levels in single particle experiments than ensemble solution samples to achieve appreciable signal-to-noise ratios. As a result, the single-particle photosensitization time and UV irradiation time were set at 30 s and 120 s. The FL intensity trajectories from more than 100 single particles were collected and analyzed. The single-particle FL data were deconvoluted to quantify the ^1^O_2_ capturing-storing and releasing abilities of the nanoarchitecture. The FL intensity of SS-MSNP shows a remarkable time-dependent increase compared to S-MSNP (Fig. 3g). The higher FL intensity of SS-MSNP than S-MSNP at any time after 532 nm photosensitization suggests that the ^1^O_2_ storing and sensing efficiency is greater when the sensor and PS are in close proximity. Conversely, the ^1^O_2_ produced by TCPP molecules in the solution is less efficiently captured and sensed by S-MSNP, presumably due to the random diffusion or the large degree of diffusion freedom for ^1^O_2_ produced in the solution. In other words, the sensors on SS-MSNP efficiently cage and sense ^1^O_2_ produced by TCPP on the same nanoarchitecture. Fig. 3h shows the UV-induced ^1^O_2_-releasing abilities of S-MSNP and SS-MSNP single particles. Both systems show temporally controlled ^1^O_2_ release. Also, we examined the photostability of S-MSNPs and SS-MSNPs under UV or 532 nm excitation (Fig. S6†). SS-MSNPs excited with the 532 nm laser in the presence of NaN_3_ showed steady FL at 440 or 650 nm, indicating both the sensor and TCPP remain stable. Also, S-MSNP or SS-MSNP excited with 365 nm light showed steady FL, suggesting low photodimerization efficiency for the sensors covalently conjugated to the Si NPs. Therefore, the SS-MSNP acts as a nanoreactor that promotes the ^1^O_2_ storing and light-triggered sustained ^1^O_2_ release. These results demonstrate the potential of SS-MSNP nanoarchitecture as an efficient ^1^O_2_ sensor and reservoir for PDT, particularly for fractional PDT.

### ^1^O_2_ generation and sensing in cells

**Fig. 4:**
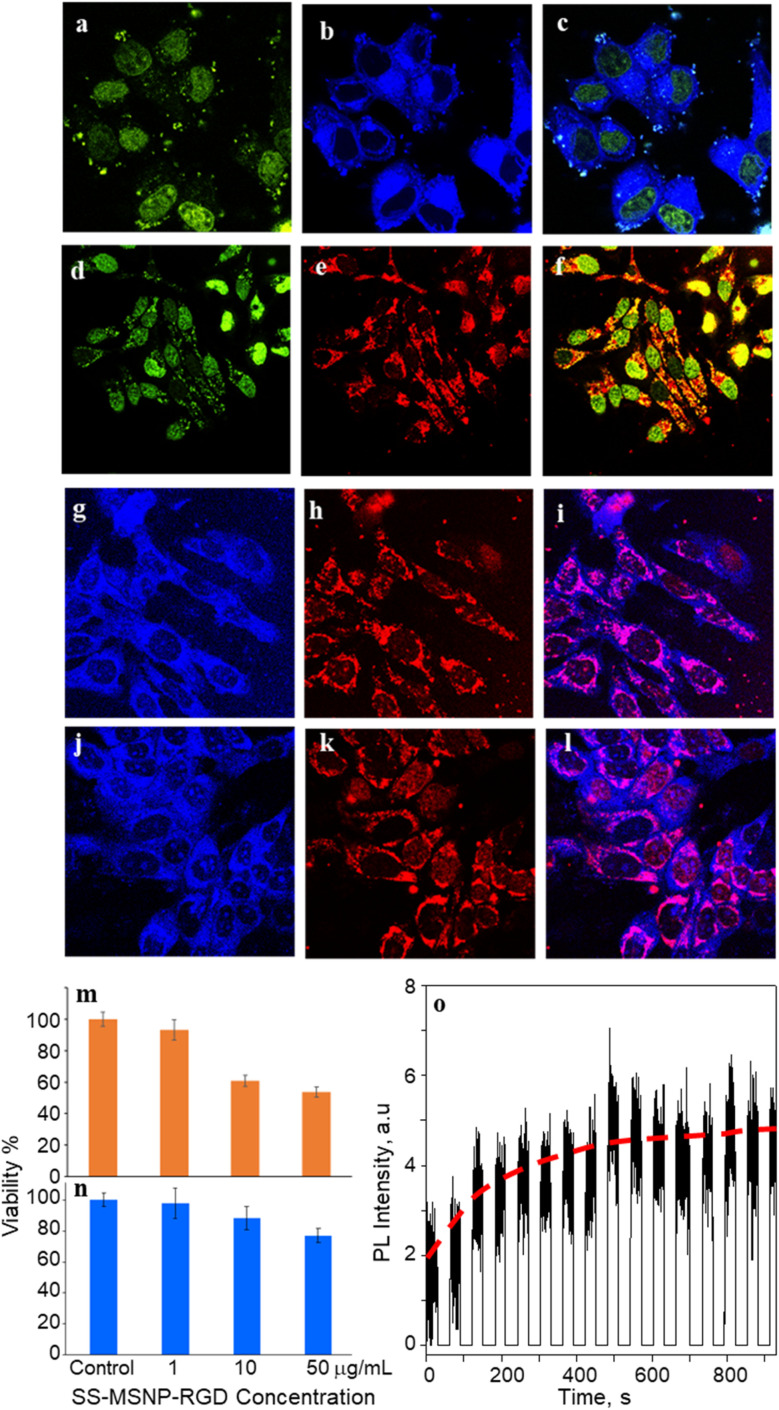
Intracellular imaging and ^1^O_2_ detection. Fluorescence (FL) images of cells labeled with 50 μg mL^−1^ of (a–c) S-MSNP, (d–f) TCPP-MSNP, (g–i) SS-MSNP and (j–l) SS-MSNP-RGD conjugates showing FL from the (b, g, and j) sensor, (a and d), Syto 16, (e, h, and k) TCPP, (c, f, i, and l) overlaid images. (m and n) MTT cell viability histograms for MCF cells treated with different concentrations of SS-MSNP-RGD assembly under (m) 500–650 nm band-pass filtered light irradiation (10 mW cm^−2^) for 30 min, and (n) dark. (o) An intracellular single-particle FL intensity trajectory for MCF7 cells incubated with SS-MSNP-RGD conjugate and photosensitized with a 532 nm laser. The FL intensity was recorded every 30 s after the photosensitization. The images are (a–c, g–l) 150 × 150 μm^2^ and (d–f) 200 × 200 μm^2^.

We investigated the bioimaging imaging and PDT potentials of the sensor- and sensitizer-conjugated MSNPs. First, we treated the conjugates with the breast cancer cell MCF7. Fig. 4a–i shows FL images of the cells incubated with S-MSNP, TCPP-MSNP, SS-MSNP, or SS-MSNP-RGD for 1 h under ambient conditions. Also, the cells were stained with the nucleus staining dye Syto 16 (green FL in Fig. 4a, d, and f). The blue FL of S-MSNPs distributed in the cytoplasm indicates nonspecific endocytosis of the NPs. The appreciably intense intracellular blue FL suggests blockage of PIET in the sensor by endogenous ^1^O_2_, pointing towards the localization of S-MSNPs to ^1^O_2_-rich domains, such as the mitochondria. Fig. 4d–f shows FL images of the cells labeled with Syto 16 and the TCPP-MSNP conjugate. The intracellular red FL shows the effective uptake of TCPP-MSNP by MCF7 cells. Similarly, cells labeled with SS-MSNPs showed intracellular blue FL (Fig. 4g) of the oxidized sensor and red FL (Fig. 4h) of TCPP. Although we do not rule out the role of multiple COOH groups in TCPP on the endocytosis of TCPP-MSNP or SS-MSNP, the primary amino groups introduced during the silanization step (Fig. 1c) are common to S-MSNP, TCPP-MSNP, and SS-MSNP, which should have a role in the cellular uptake of these NPs.

Despite the nonspecific intracellular delivery of SS-MSNP, we applied the RGD-conjugated SS-MSNPs (SS-MSNP-RGD) to MCF7 cells to improve the cellular uptake and to understand the role of α_v_β_3_ integrin-mediated endocytosis of the conjugate.^70^Fig. 4j–l shows the FL images of cells incubated with the SS-MSNP-RGD conjugate. The intense intracellular blue and red FL suggests efficient cellular uptake of the conjugate. Nevertheless, cells preincubated with the (arginine)_8_ (R8) peptide that blocks α_v_β_3_ integrin and treated with SS-MSNP-RGD showed appreciable intracellular blue and red FL, suggesting α_v_β_3_ integrin-mediated endocytosis is not the only intracellular delivery mechanism. Therefore, like the conjugates without RGD (Si-TCPP, S-MSNP, SS-MSNP, and SS-MSNP; Fig. 4a–g), macropinocytosis is involved in the intracellular pathway of SS-MSNP-RGD, whereas RGD-free conjugates are delivered by macropinocytosis alone. Although multiple intracellular delivery routes are involved for SS-MSNP and SS-MSNP-RGD, a uniform and efficient intracellular NP distribution for the RGD conjugate denotes passive and active targeting for nanoparticle-guided therapy.^36^ Despite the confocal FL images showing effective accumulation of the RGD conjugate inside the cells, microtoming and cross-section TEM imaging helped us locate the NPs inside. Here, the cells cultured on 1 cm × 1 cm polypropylene plates deposited in a cell culture plate were washed with PBS, labeled with SS-MSNP-RGD for 30 min, gently washed with PBS (3 times), and dried under a vacuum. The presence of the cells on the polypropylene plates was identified by optical imaging, followed by microtoming and TEM imaging. Fig. S8† shows the cross-section TEM image of a cell treated with SS-MSNP-RGD. The dark contrast corresponds to the NPs in the subcellular compartment.

Following the cellular uptake of SS-MSNP-RGD nanoarchitecture, we examined the intracellular ^1^O_2_ generation and release by single-cell FL measurements and time-lapsed FL imaging. The cells incubated with the nanoarchitecture were photo-irradiated continuously using a 532 nm laser, and the FL trajectories were recorded for each step of the 532 nm laser and UV illuminations. The time-correlated FL intensity trajectory (Fig. 4o) suggests intracellular ^1^O_2_ generation, storing, and release, like the single particle results in Fig. 3. Further, the cell morphology changes were observed by time-lapse imaging of cells under continuous light irradiation (Fig. S9†). With time under irradiation, we detected apoptosis with corpuscles and cell shrinkage.^44^ These results confirm that ^1^O_2_ generated and released continuously in the cells accelerates cell death mainly by the apoptotic pathway.

Low dark cytotoxicity and high phototoxicity are important factors in PDT. We examined the cytotoxicity of S-MSNP and SS-MSNP-RGD to MCF7 cells. MTT cytotoxicity histograms are shown in Fig. 4m and n. We found the lethal dose (LD) for 50% cell viability (LD_50_) under the dark at ≫100 μg mL^−1^ SS-MSNP-RGD. Also, >80% cell viability was evident for the cells treated with 10 or 1 μg mL^−1^ SS-MSNP-RGD solutions in the dark (Fig. 4n). Comparable dark cell viability values were obtained for S-MSNP (Fig. S10†). Phototoxicity of SS-MSNP-RGD to MCF7 cells was evaluated under the above labeling conditions. The labeled cells were irradiated with 500–650 nm band-pass filtered light (10 mW cm^−2^) for 30 min before the MTT treatment. Phototoxicity of SS-MSNP-RGD for 1, 10, and 50 μg mL^−1^ sample solutions are shown in Fig. 4m, showing appreciable phototoxicity for 50 μg mL^−1^ (52%) and 10 μg mL^−1^ (42%) samples. The phototoxicity is attributed to ^1^O_2_ generation by the sensitizer in the NPs, which is consistent with the ensemble and single-particle spectroscopic and microscopic data.

**Fig. 5:**
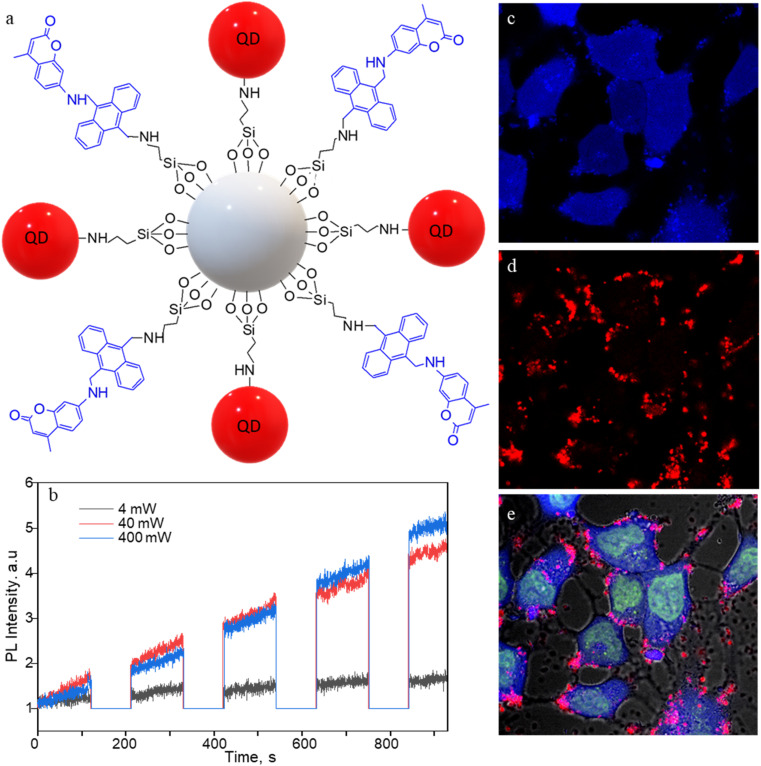
CdSe/ZnS QD- and sensor-conjugated MSNPs (S-QD-MSNP) for ^1^O_2_ generation and intracellular imaging. (a) The structure of S-QD-MSNP nanoarchitecture. (b) FL intensity trajectories (440 nm) showing ^1^O_2_ production by QDs under 532 nm laser irradiation (60 s) and UV illumination (120 s) sequence at different photosensitization powers. (c–e) FL images of MCF7 cells labeled with SYTO-16 (5 μM solution) and the S-QD-MSNP nanoarchitecture (50 μg mL^−1^) showing the emission from the (c) sensor, (d) QD655, and (e) the overlaid image. The photosensitization and ^1^O_2_ release are temporally separated by 30 s shutter. The images are 150 × 150 μm^2^.

Apart from SS-MSNP-RGD, we used NIR-emitting CdSe/ZnS QD for testing ^1^O_2_ production and sensing in cells. QDs have been widely employed in PDT and cell/tissue imaging experiments in cell/animal models due to deep tissue penetration, improved water solubility, and biocompatibility.^53–55^ Recently, Vetrone and coworkers demonstrated a multifunctional MSNP theranostic integrated with QDs, Fe_3_O_4_, and DOX for combined bimodal (NIR-fluorescence and magnetic resonance) imaging, drug delivery, hyperthermia, and phototherapy.^55^ Also, the ability of QDs to produce ROS makes them promising for PDT.^56^ Therefore, we prepared MSNPs conjugated with the sensor, CdSe/ZnS QDs (Fig. 5a), and evaluated the ^1^O_2_ production of the S-QD-MSNP nanoarchitecture by photosensitizing at 532 nm with different laser power densities. In Fig. 5b, the FL intensity trajectories recorded by 60 s 532 nm irradiation, followed by 120 s UV illumination, showed appreciable FL (440 nm, coumarin) intensity increases at high excitation power (≥40 mW cm^−2^). The high excitation intensity requirement is due to the low ^1^O_2_ quantum yield of QDs.^57,58^ The ^1^O_2_ generation and trapping behavior of the S-QD-MSNP at the ensemble level also showed a similar trend (Fig. S7†), in agreement with the single-particle studies. Therefore, the sensor is promising to prepare sensor-sensitizer systems using different nanoparticle and molecular photosensitizers.

### *In vivo* experiments

We examined the *in vivo* bioimaging potential of SS-MSNP-RGD in B6 mice subcutaneously and intravenously. First, we examined and optimized the fluorescence of the samples using a small-animal imaging system by applying different band-pass filters and excitation light sources. Fig. S11† shows the bright-field and FL images of S-MSNP, SS-MSNP, and SS-MSNP-RGD samples. S-MSNP showed blue-green fluorescence, of which the long wavelength part of the coumarin dye was collected due to the small Stoke's shift (*λ*_ex_ = 445–490 nm band-pass filtered light, and the FL was collected through a 515 nm LP filter). Fig. S11c and d† shows the emission from the sensitizer in TCPP-MSNP and SS-MSNP-RGD samples. Subsequently, we examined the images of the B6 mice subcutaneously injected with the TCPP-MSNP or SS-MSNP-RGD samples. The characteristic FL (>670 nm) of the sensitizer (Fig. S11e and f†) was detected, which efficiently penetrated the skin tissue. Conversely, the FL from the sensor was not detected in mice subcutaneously or intravenously injected with S-MSNP or SS-MSNP samples, which is due to the poor penetration of the sensor FL (*ca.* 440 nm) through the skin/tissue and the overlapping sensor FL with the mice autofluorescence.

We found higher FL intensities in the mice liver 1 to 24 h post intravenous injection of the S-MSNP or SS-MSNP-RGD conjugates (Fig. S11i–l†). The FL signals correspond to 670 nm or longer wavelengths solely from the sensitizer parts. Conversely, the sensor FL was not detected even when the SS-MSNP-RGD sample-injected mice were exposed to 532 nm light or an SS-MSNP-RGD sample was photosensitized at 532 nm for 30 min before the injection. This absence of the blue signals is attributed to its overlapping with the tissue autofluorescence or its reabsorption by tissues. The 670 nm FL intensity in the liver of the mice injected with the TCPP-MSNP was increased within 24 h post-injection (Fig. S11k†), showing a gradual accumulation. Conversely, for a mouse injected with SS-MSNP-RGD, the FL from the liver was decreased within 24 h (Fig. S11l†) with an increase in the FL intensity in the bladder. These observations suggest that the RGD conjugation helps to circulate and excrete the NPs. Further imaging, toxicity, and pharmacokinetic investigations are necessary to assess the bioimaging or phototherapeutic potentials of the above sensor, sensitizer, or nanoparticles. Also, NIR-emitting sensors and sensitizers are needed for *in vivo* applications.

## Summary

We constructed a fluorescence turn-on photosensitizer-sensor-peptide nanoarchitecture for intracellular ^1^O_2_ generation, storing, controlled release, and sensing. The on-demand ^1^O_2_ sensing-releasing-assisted fluorescence intensity enhancement and fluorescence color change of this architecture were uncovered by fluorescence measurements of solutions, living cells, or single particles. The small size of the nanoarchitecture, within the ^1^O_2_ diffusion length, promoted tandem photo-triggered fluorescence switching and enabled bimodal fluorescence cell imaging activated by on-demand intracellular sensor oxidation and ^1^O_2_ release. Under continuous laser irradiation, ^1^O_2_-induced cell death was also observed. The MTT assay results revealed low dark toxicity and high phototoxicity of the sensor-sensitizer-silica-peptide conjugates to MCF7 cells. Also, preliminary *in vivo* studies on B6 mice intravenously or subcutaneously injected with the conjugates help elucidate the *in vivo* application of the conjugate. Nevertheless, the blue emission from the sensor is masked by reabsorption and the tissue autofluorescence, suggesting the significance of developing sensors and sensitizers absorbing and emitting in the NIR biological window.

## Data availability

Data supporting the findings of this study are available within the article ESI.†

## Author contributions

V. B. and P. W. conceived the idea. V. B. led the project. J. S. conducted the chemical synthesis, bioconjugate reactions, fluorescence detection studies, and cell imaging. K. O. and M. S. conducted the *in vivo* experiment. T. O. performed STEM and microtoming experiments. J. S. wrote the manuscript draft. All authors contributed to the copy-editing of the manuscript. J. S., P. W. and V. B. finalized the manuscript.

## Conflicts of interest

The authors declare no conflict of interest.

## Supplementary Material

SC-015-D3SC03877G-s001

## References

[cit1] DeRosa M. C., Crutchley R. J. (2002). Coord. Chem. Rev..

[cit2] Yang W., Zhang F., Deng H., Lin L., Wang S., Kang F., Yu G., Lau J., Tian R., Zhang M., Wang Z., He L., Ma Y., Niu G., Hu S., Chen X. (2020). ACS Nano.

[cit3] Xiao Y. F., Chen W. C., Chen J. X., Lu G., Tian S., Cui X., Zhang Z., Chen H., Wan Y., Li S., Lee C. S. (2022). ACS Appl. Mater. Interfaces.

[cit4] Liu K., Liu X., Zeng Q., Zhang Y., Tu L., Liu T., Kong X., Wang Y., Cao F., Lambrechts S. A. G., Aalders M. C. G., Zhang H. (2012). ACS Nano.

[cit5] Zhao N., Ding B., Zhang Y., Klockow J. L., Lau K., Chin F. T., Cheng Z., Liu H. (2020). J. Control. Release.

[cit6] Cao Z., Li D., Wang J., Yang X. (2021). Acta Biomater..

[cit7] Filatov M. A., Karuthedath S., Polestshuk P. M., Savoie H., Flanagan K. J., Cy C., Sitte E., Telitchko M., Laquai F., Boyle R. W., Senge M. O. (2017). J. Am. Chem. Soc..

[cit8] De Bonfils P., Verron E., Nun P., Coeffard V. (2021). ChemPhotoChem.

[cit9] Chen B., Yang Y., Wang Y., Yan Y., Wang Z., Yin Q., Zhang Q., Wang Y. (2021). ACS Appl. Mater. Interfaces.

[cit10] Chen T., Hou P., Zhang Y., Ao R., Su L., Jiang Y., Zhang Y., Cai H., Wang J., Chen Q., Song J., Lin L., Yang H., Chen X. (2021). Angew. Chem., Int. Ed..

[cit11] Lam T. L., Tong K. C., Yang C., Kwong W. L., Guan X., De Li M., Kar-Yan Lo V., Lai-Fung Chan S., Lee Phillips D., Lok C. N., Che C. M. (2019). Chem. Sci..

[cit12] Liang D., Zhang Y., Wu Z., Chen Y. J., Yang X., Sun M., Ni R., Bian J., Huang D. (2018). Sensors Actuators, B.

[cit13] Yuan Z., Yu S., Cao F., Mao Z., Gao C., Ling J. (2018). Polym. Chem..

[cit14] Bresolí-Obach R., Nos J., Mora M., Sagristà M. L., Ruiz-González R., Nonell S. (2016). Methods.

[cit15] Sun M., Krishnakumar S., Zhang Y., Liang D., Yang X., Wong M. W., Wang S., Huang D. (2018). Sensors Actuators, B.

[cit16] Liu X., Dai P., Gu T., Wu Q., Wei H., Liu S., Zhang K. Y., Zhao Q. (2020). J. Inorg. Biochem..

[cit17] Zhu J., Zou J., Zhang J., Sun Y., Dong X., Zhang Q. (2019). J. Mater. Chem. B.

[cit18] Deng J., Liu F., Wang L., An Y., Gao M., Wang Z., Zhao Y. (2019). Biomater. Sci..

[cit19] Chen J., Li D., Huo B., Zhang F., Zhao X., Yuan G., Chen D., Song M., Xue J. (2019). Mol. Pharm..

[cit20] Wang D., Xue B., Ohulchanskyy T. Y., Liu Y., Yakovliev A., Ziniuk R., Xu M., Song J., Qu J., Yuan Z. (2020). Biomaterials.

[cit21] Chen T., Hou P., Zhang Y., Ao R., Su L., Jiang Y., Zhang Y., Cai H., Wang J., Chen Q., Song J., Lin L., Yang H., Chen X. (2021). Angew. Chem., Int. Ed..

[cit22] Lv W., Cao M., Liu J., Hei Y., Bai J. (2021). Acta Biomater..

[cit23] Fan J., Yang M., Zhang J., Shabat D., Peng X. (2020). ACS Sensors.

[cit24] Yang D. C., Wang S., Weng X. L., Zhang H. X., Liu J. Y., Lin Z. (2021). ACS Appl. Mater. Interfaces.

[cit25] Deng K., Wu B., Wang C. X., Wang Q., Yu H., Li J. M., Li K. H., Zhao H. Y., Huang S. W. (2020). Adv. Healthcare Mater..

[cit26] Lai H., Yan J., Liu S., Yang Q., Xing F., Xiao P. (2020). Angew. Chem., Int. Ed..

[cit27] Liu Q., Tian J., Tian Y., Sun Q., Sun D., Wang F., Xu H., Ying G., Wang J., Yetisen A. K., Jiang N. (2021). ACS Nano.

[cit28] Wang D., Wu H., Phua S. Z. F., Yang G., Qi Lim W., Gu L., Qian C., Wang H., Guo Z., Chen H., Zhao Y. (2020). Nat. Commun..

[cit29] Kv R., Liu T. I., Lu I. L., Liu C. C., Chen H. H., Lu T. Y., Chiang W. H., Chiu H. C. (2020). J. Control. Release.

[cit30] Ke X., Wang D., Chen C., Yang A., Han Y., Ren L., Li D., Wang H. (2014). Nanoscale Res. Lett..

[cit31] Gangopadhyay M., Jana A., Rajesh Y., Bera M., Biswas S., Chowdhury N., Zhao Y., Mandal M., Singh N. D. P. (2016). ChemistrySelect.

[cit32] Wan Y., Lu G., Wei W. C., Huang Y. H., Li S., Chen J. X., Cui X., Xiao Y. F., Li X., Liu Y., Meng X. M., Wang P., Xie H. Y., Zhang J., Wong K. T., Lee C. S. (2020). ACS Nano.

[cit33] Farooq S., de Araujo R. E. (2021). High Performance Gold Nanoshells for Singlet Oxygen Generation Enhancement. Photodiagn. Photodyn. Ther..

[cit34] Mendoza C., Emmanuel N., Páez C. A., Dreesen L., Monbaliu J. C. M., Heinrichs B. (2018). ChemPhotoChem.

[cit35] Kohle F. F. E., Li S., Turker M. Z., Wiesner U. B. (2020). ACS Biomater. Sci. Eng..

[cit36] Liu R., Gao Y., Liu N., Suo Y. (2021). Photodiagn. Photodyn. Ther..

[cit37] Kim J., Lee H., Lee J. Y., Park K. H., Kim W., Lee J. H., Kang H. J., Hong S. W., Park H. J., Lee S., Lee J. H., Park H. D., Kim J. Y., Jeong Y. W., Lee J. (2020). Appl. Catal., B.

[cit38] Liu X., Jiang J., Chang C. H., Liao Y. P., Lodico J. J., Tang I., Zheng E., Qiu W., Lin M., Wang X., Ji Y., Mei K. C., Nel A. E., Meng H. (2021). Small.

[cit39] Meng H., Wang M., Liu H., Liu X., Situ A., Wu B., Ji Z., Chang C. H., Nel A. E. (2015). ACS Nano.

[cit40] Liu X., Jiang J., Liao Y. P., Tang I., Zheng E., Qiu W., Lin M., Wang X., Ji Y., Mei K. C., Liu Q., Chang C. H., Wainberg Z. A., Nel A. E., Meng H. (2021). Adv. Sci..

[cit41] Liu X., Jiang J., Chan R., Ji Y., Lu J., Liao Y. P., Okene M., Lin J., Lin P., Chang C. H., Wang X., Tang I., Zheng E., Qiu W., Wainberg Z. A., Nel A. E., Meng H. (2019). ACS Nano.

[cit42] Das M., Solanki A., Ganesh A., Thakore S. (2021). Photodiagn. Photodyn. Ther..

[cit43] Tarn D., Ashley C. E., Xue M. I. N., Carnes E. C., Zink J. I., Brinker C. J. (2013). Acc. Chem. Res..

[cit44] Dogra P., Adolphi N. L., Wang Z., Lin Y.-S., Butler K. S., Durfee P. N., Croissant J. G., Noureddine A., Coker E. N., Bearer E. L., Cristini V., Brinker C. J. (2018). Nat. Commun..

[cit45] Jiao L., Zhang X., Cui J., Peng X., Song F. (2019). ACS Appl. Mater. Interfaces.

[cit46] Jiao L., Song F., Zhang B., Ning H., Cui J., Peng X. (2017). J. Mater. Chem. B.

[cit47] Jiao L., Liu Y., Zhang X., Hong G., Zheng J., Cui J., Peng X., Song F. (2020). ACS Cent. Sci..

[cit48] Yang C., Su M., Luo P., Liu Y., Yang F., Li C. A. (2021). Small.

[cit49] He Y. Q., Fudickar W., Tang J. H., Wang H., Li X., Han J., Wang Z., Liu M., Zhong Y. W., Linker T., Stang P. J. (2020). J. Am. Chem. Soc..

[cit50] Kim S., Tachikawa T., Fujitsuka M., Majima T. (2014). J. Am. Chem. Soc..

[cit51] Gollmer A., Arnbjerg J., Blaikie F. H., Pedersen B. W., Breitenbach T., Daasbjerg K., Glasius M., Ogilby P. R. (2011). Photochem. Photobiol..

[cit52] Liu S., Hu Y., Hu C., Xiong Y., Duan M. (2019). J. Porphyrins Phthalocyanines.

[cit53] Wolfbeis O. S. (2015). Chem. Soc. Rev..

[cit54] Shibu E. S., Ono K., Sugino S., Nishioka A., Yasuda A., Shigeri Y., Wakida S. I., Sawada M., Biju V. (2013). ACS Nano.

[cit55] Yang F., Skripka A., Tabatabaei M. S., Hong S. H., Ren F., Huang Y., Oh J. K., Martel S., Liu X., Vetrone F., Ma D. (2019). Chem. Mater..

[cit56] Yaghini E., Pirker K. F., Kay C. W., Seifalian A. M., MacRobert A. J. (2014). Small.

[cit57] Pinaud F., King D., Moore H. P., Weiss S. (2004). J. Am. Chem. Soc..

[cit58] Yamashita S., Hamada M., Nakanishi S., Saito H., Nosaka Y., Wakida S., Biju V. (2015). Angew. Chem., Int. Ed..

[cit59] Vallecorsa P., Di Venosa G., Ballatore M. B., Ferreyra D., Mamone L., Sáenz D., Calvo G., Durantini E., Casas A. (2021). BMC Cancer.

[cit60] Dai Z., Liang X., Chen M., Bhattarai P., Hameed S. (2020). ACS Nano.

[cit61] Yang L., Zhou J., Wang Z., Li H., Wang K., Liu H., Wu F. (2020). Dye. Pigment..

[cit62] Xie B. R., Yu Y., Liu X. H., Zeng J. Y., Zou M. Z., Li C. X., Zeng X., Zhang X. Z. A. (2021). Biomaterials.

[cit63] Silva L. B., Castro K. A. D. F., Botteon C. E. A., Oliveira C. L. P., da Silva R. S., Marcato P. D. (2021). Front. Bioeng. Biotechnol..

[cit64] Montaseri H., Kruger C. A., Abrahamse H. (2020). Int. J. Mol. Sci..

[cit65] Gao Y., Gao D., Shen J., Wang Q. (2020). Front. Chem..

[cit66] Kohara R., Yuyama K., Shigeri Y., Biju V. (2017). ChemPhotoChem.

[cit67] Fidder H., Lauer A., Freyer W., Koeppe B., Heyne K. (2009). J. Phys. Chem. A.

[cit68] Nathusius M., Sleeman D., Pan J., Rominger F., Freudenberg J., Bunz U. H. F., Müllen K. (2021). Chem.–Eur. J..

[cit69] Fudickar W., Linker T. (2012). J. Am. Chem. Soc..

[cit70] Manzanares D., Ceña V. (2020). Pharmaceutics.

