# Supplementary material for: Photosensitizer-singlet oxygen sensor conjugated silica nanoparticles for photodynamic therapy and bioimaging
Source: Chem Sci. 2023 Dec 14;15(6):2007–18. doi: 10.1039/d3sc03877g (PMC10848760; doi:10.1039/d3sc03877g)
Supplement: SC-015-D3SC03877G-s001 [file SC-015-D3SC03877G-s001.pdf]

Electronic Supplementary Material (ESI) for Chemical Science.
This journal is © The Royal Society of Chemistry 2023

Supporting Information for

Photosensitizer-Singlet Oxygen Sensor Conjugated Silica Nanoparticles for Photodynamic Therapy and Bioimaging

Jeladhara Sobhanan,[1,2] Kenji Ono,[3] Takuya Okamoto,[1,4] Makoto Sawada,[3] Paul S.Weiss,[5] Vasudevanpillai Biju*,[1,4]

[1]Graduate School of Environmental Science, Hokkaido University, Sapporo, Hokkaido 060-0810, Japan

[2] Department of Chemistry, Rice University, Houston, Texas 77005, USA

[3]Research Institute of Environmental Medicine, Nagoya University, Nagoya 464-8601, Japan

[4]Research Institute for Electronic Science, Hokkaido University, Sapporo, Hokkaido 001-0020, Japan

[5]California NanoSystems Institute and the Departments of Chemistry and Biochemistry, Bioengineering, and Materials Science and Engineering, University of California Los Angeles, CA 90095-1487, USA

Zeta potential measurements

We used the instrument ELS-Z 2 (Otsuka Electronics Co., Ltd.) for the zeta potential measurements. The samples were taken in a flow cell unit, and the measurements were by the laser Doppler method at 25 °C. The average electric field was ca. 14 V/cm. We calculated the zeta potentials by the Smoluchowski method. Each sample was measured three times with five points each (0.15, 0.325, 0.5, 0.675, 0.85 mm from the bottom of the cuvette). The samples were prepared in the PBS buffer. The zeta potential values are +16.91 mV for S-MNSP, +15.52 mV for SS-MSNP, and -15.02 for SS-MNP-RGD.

DLS measurements.

We estimated the sizes of SS-MSNP and SS-MSNP particles by DLS measurements. The sample concentrations were set at 15 nM, and the data were collected at 25 °C. The average sizes (Figure S1) of the particles are 179 nm (SS-MSNP) and 192 nm (SS-MSNP), which are comparable to the size of the parent silica particles as observed in the SEM image (Figure 1).

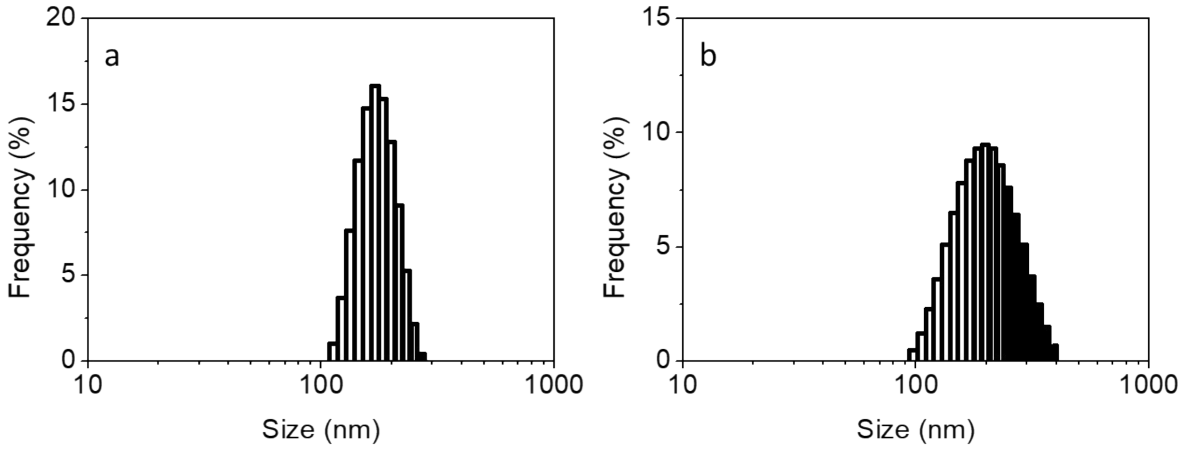

Figure S1. The Size distributions of SS-MSNP and SS-MSNP particles by DLS measurements.

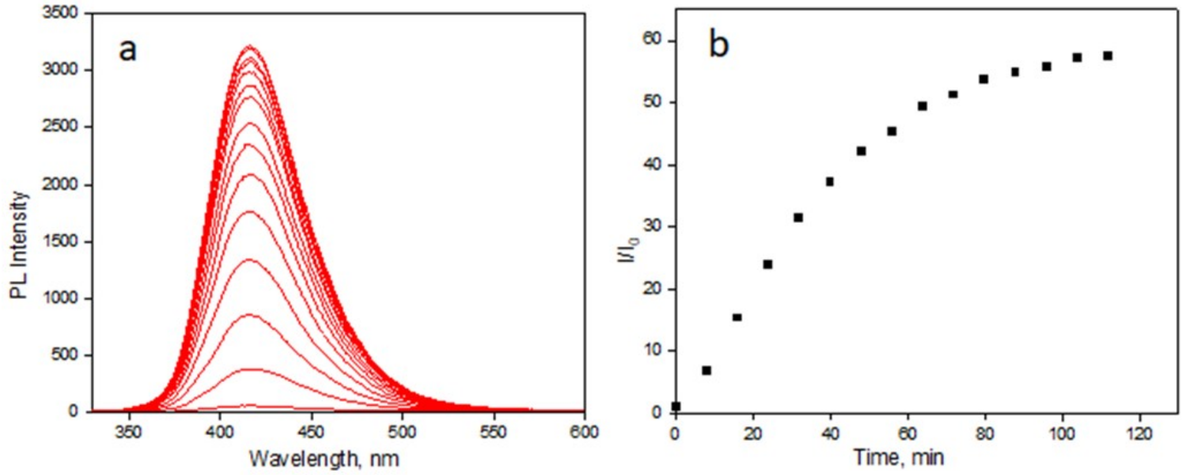

Figure S2. (a) Fluorescence (λ_{ex}=320 nm) spectra of a solution of **4** (10 μM) and NaN_3 (150 μM) in CH_3CN before and after UV illumination (365 nm) for 112 min; each spectrum was recorded after 5 min photoirradiation, (b) Time-trace of the peak fluorescence intensities in 'a'.

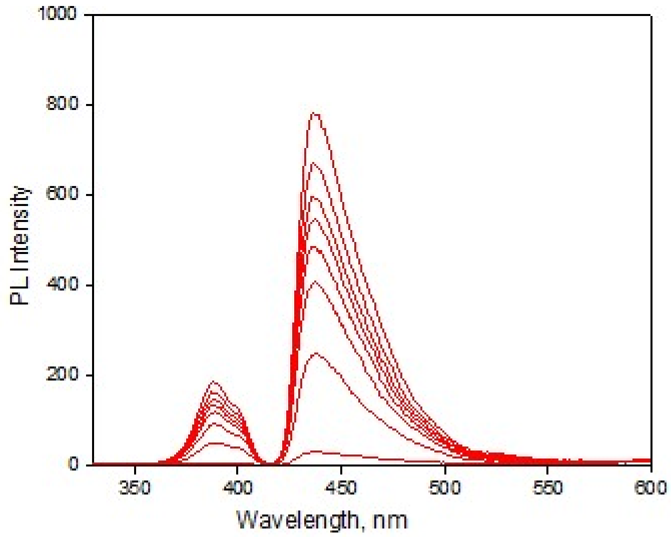

Figure S3. Fluorescence (λ_{ex}=320 nm) spectra of solution of **4** (10 μM) and TCPP (5 μM) in CH_3CN before and after photoactivation at 532 nm (50 mW cm^{-2}) for 70 min at 10 min intervals.

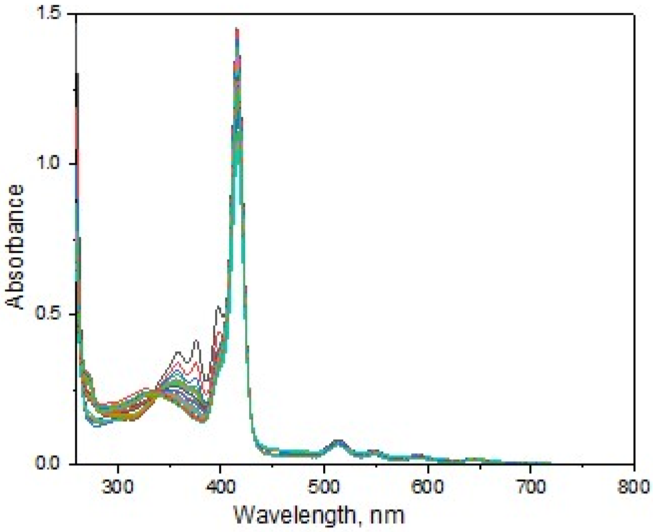

Figure S4. Absorption spectra of a solution of **4** (10 μM) and TCPP (5 μM) in CH_3CN before and after photoactivation at 532 nm (50 mW cm^{-2}) for 30 min followed by UV illumination (365 nm) for 100 min, each spectrum was recorded after 5 min photoirradiation.

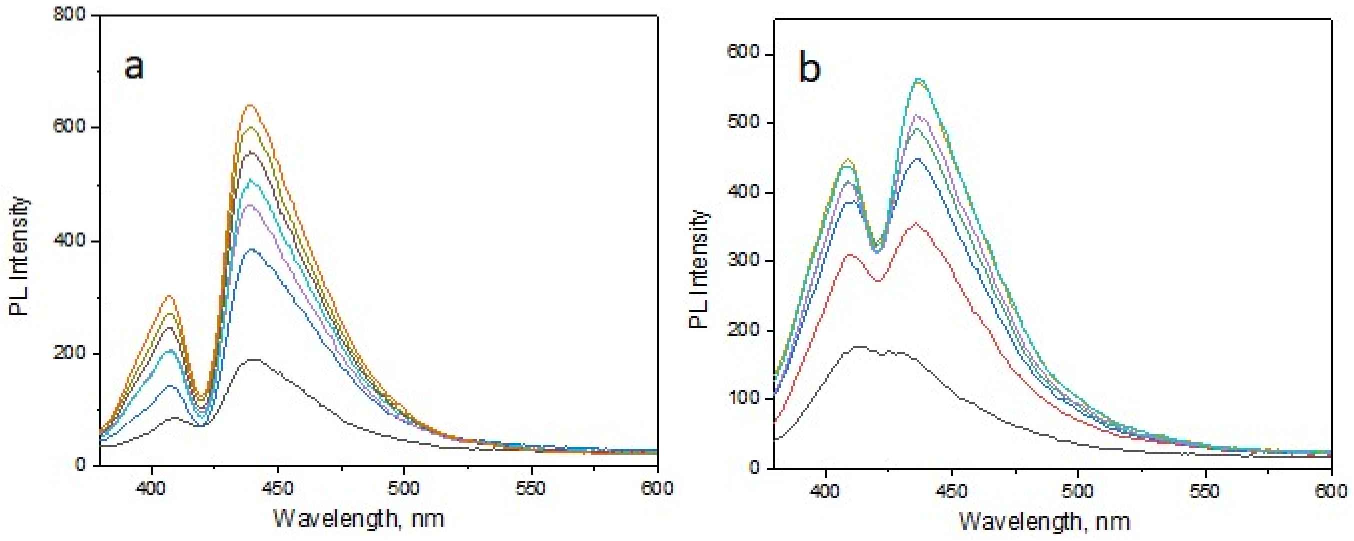

Figure S5. Fluorescence (λ_{ex}=320 nm) spectra of (a) S-MSNPs in a TCPP solution (5 μM) and (b) an SS-MSNP solution in DMF before and after photoactivation at 532 nm (50 mW cm^{-2}) for 60 min. The spectra were recorded at 10 min intervals of photoirradiation.

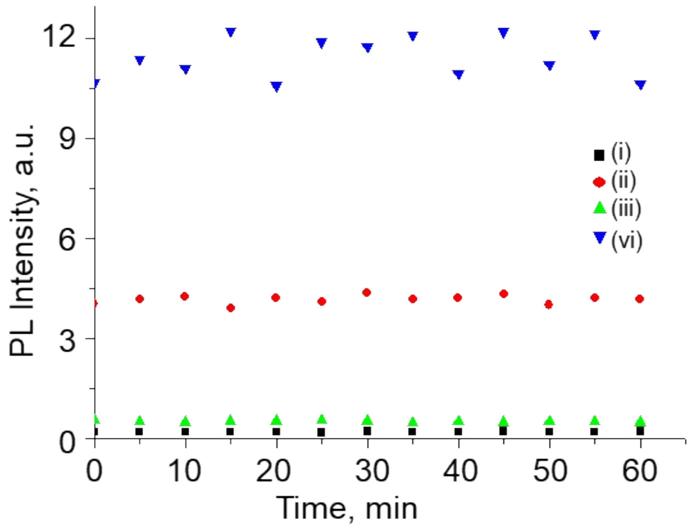

Figure S6. Photostability of S-MSNPs and SS-MSNP. (i) The background level for S-MSNP, (ii) the PL of S-MSNP, (iii) the background level of SS-MSNP, and (iv) the PL of SS-MSNP. The samples were irradiated at (ii) 365 nm (5 mW cm^{-2}, S-MSNP) and (iv) 532 nm (5 mW cm^{-2}, SS-MSNP). The intensities were collected through a 420 nm long-pass filter (S-MSPN) or a 640 nm long-pass filter (SS-MSNP).

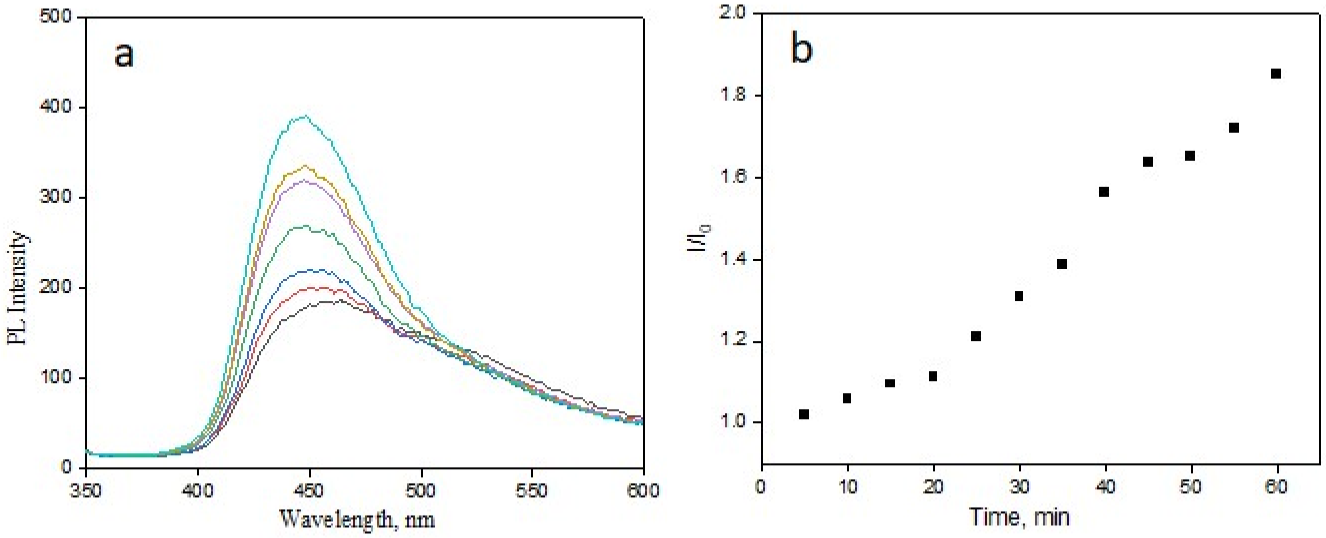

Figure S7. (a) Fluorescence (λ_{ex}=320 nm) spectra of a solution of S-QD-MSNPs before and after photoactivation at 532 nm (50 mW cm^{-2}) for 60 min, and (b) time-trace of the peak fluorescence intensities in 'a'.

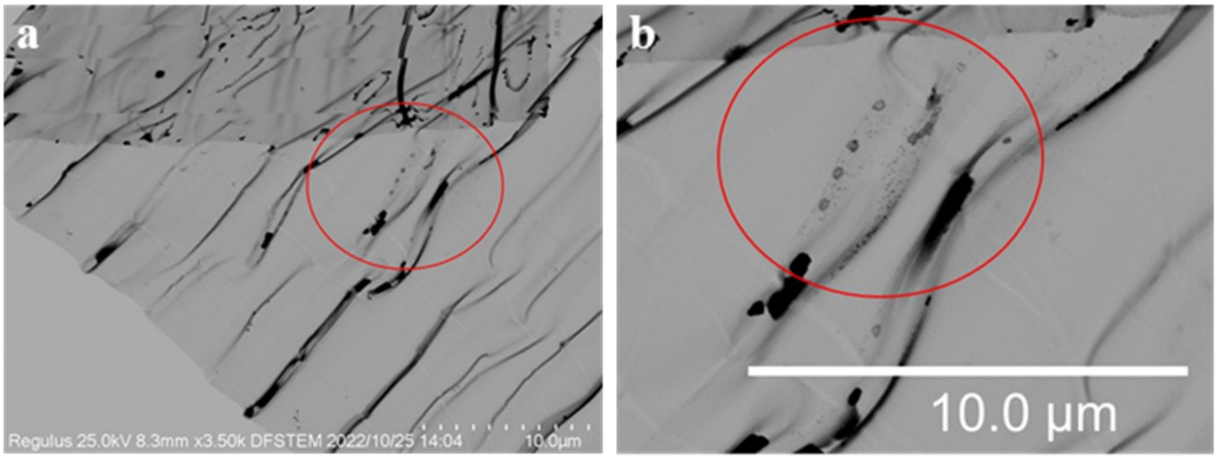

Figure S8. (a,b) Dark-field and (c) zoomed-in bright-field STEM images of MCF7 cells treated with SS-MSNP-RGD. The images were recorded after critical drying and microtoming of the cells incubated with SS-MSNP-RGD.

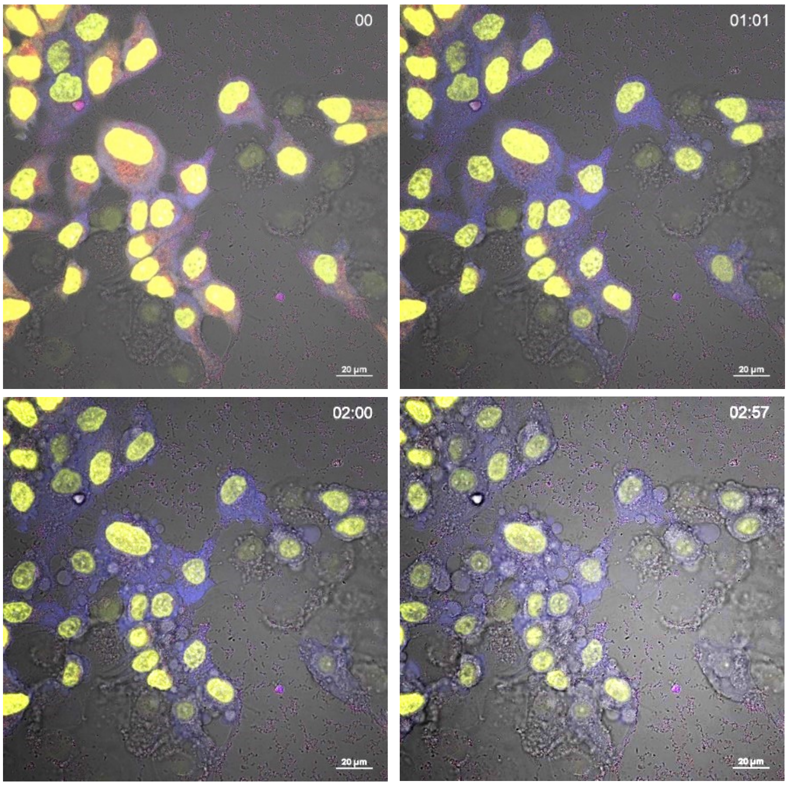

Figure S9. Time-lapse images of MCF7 cells incubated with the SS-MSNP-RGD conjugate and Syto 16 for 30 min under continuous light irradiation.

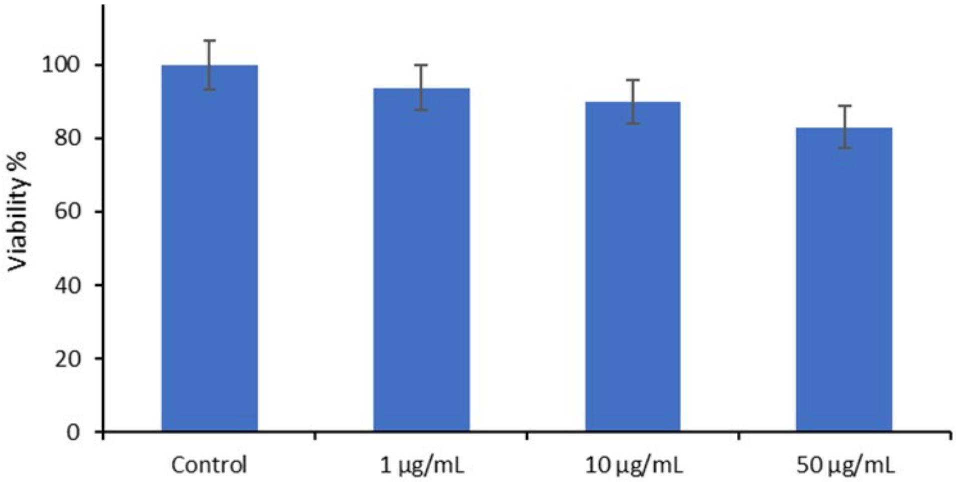

Figure S10. MTT cell viability histogram for MCF7 cells treated with different concentrations of S-MSNP samples.

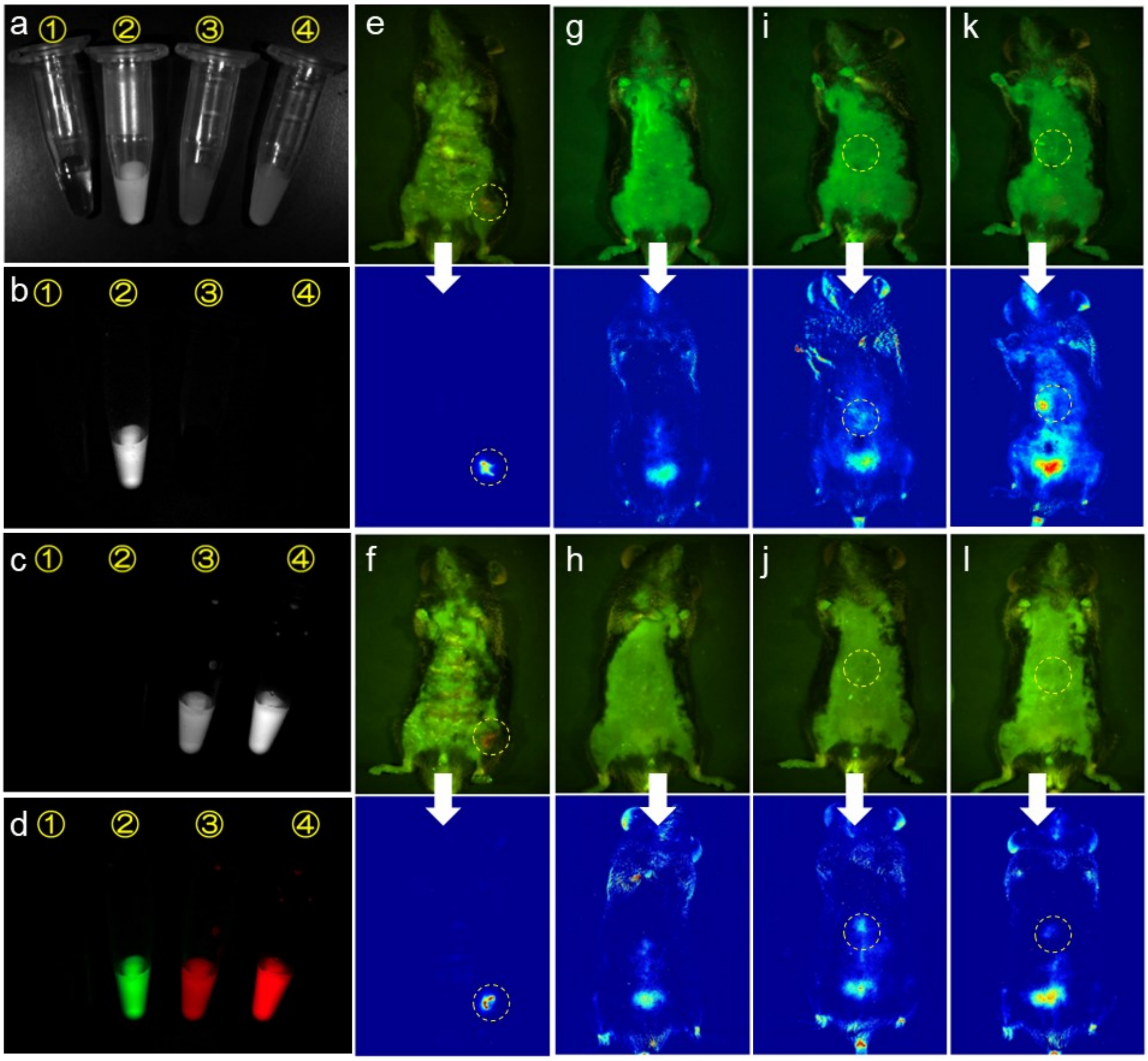

Figure S11. Optical images of samples and B6 mice. (a-d) bright-field/FL images of ① PBS (negative control), ②S-MSNP, ③SS-MSNP, and ④ SS-MSNP-RGD. (e-l) FL images of B6 mice (e) subcutaneously injected with a 200 μL (2 mg) TCPP-MSNP conjugate solution in PBS, (f) subcutaneously injected with a 200 μL (2 mg) SS-MSNP-RGD solution in PBS, (i) intravenously injected with a 200 μL (2 mg) S-MSNP solution in PBS and acquired at 1 h post-injection, (j) intravenously injected with a 200 μL (2 mg) SS-MSNP-RGD solution in PBS and acquired at 1 h post-injection, (k) the image of the mouse in 'i' obtained 24 h post-injection, (l) the image of the mouse in 'j' obtained 24 h post-injection. (g, h) Pre-injection images. The excitation source was 445-490 nm band-pass filtered light, and the FL was collected through a 515 nm LP filter.
